# Advanced Bioinformatics Analysis and Genetic Technologies for Targeting Autophagy in Glioblastoma Multiforme

**DOI:** 10.3390/cells12060897

**Published:** 2023-03-15

**Authors:** Amanda J. Manea, Swapan K. Ray

**Affiliations:** Department of Pathology, Microbiology, and Immunology, University of South Carolina School of Medicine, 6439 Garners Ferry Road, Columbia, SC 29209, USA

**Keywords:** autophagy inhibition, bioinformatics analysis, CRISPR-Cas9 gene editing, glioblastoma multiforme, mRNAs, novel combination therapy option

## Abstract

As the most malignant primary brain tumor in adults, a diagnosis of glioblastoma multiforme (GBM) continues to carry a poor prognosis. GBM is characterized by cytoprotective homeostatic processes such as the activation of autophagy, capability to confer therapeutic resistance, evasion of apoptosis, and survival strategy even in the hypoxic and nutrient-deprived tumor microenvironment. The current gold standard of therapy, which involves radiotherapy and concomitant and adjuvant chemotherapy with temozolomide (TMZ), has been a game-changer for patients with GBM, relatively improving both overall survival (OS) and progression-free survival (PFS); however, TMZ is now well-known to upregulate undesirable cytoprotective autophagy, limiting its therapeutic efficacy for induction of apoptosis in GBM cells. The identification of targets utilizing bioinformatics-driven approaches, advancement of modern molecular biology technologies such as clustered regularly interspaced short palindromic repeats (CRISPR)—CRISPR-associated protein (Cas9) or CRISPR-Cas9 genome editing, and usage of microRNA (miRNA)-mediated regulation of gene expression led to the selection of many novel targets for new therapeutic development and the creation of promising combination therapies. This review explores the current state of advanced bioinformatics analysis and genetic technologies and their utilization for synergistic combination with TMZ in the context of inhibition of autophagy for controlling the growth of GBM.

## 1. Introduction

Glioblastoma multiforme (GBM) is the most common and aggressive primary malignant brain tumor, with a median patient survival of less than 15 months from the time of diagnosis [1]. Classified as a Grade IV neoplasm by the World Health Organization (WHO), GBM is histologically characterized by elevated levels of mitotic activity, loss of common morphological characteristics of mature cells, cellular pleomorphism, abnormal appearance of nuclei, coagulation necrosis, and high vascular proliferation combined with intra-tumoral and inter-tumoral heterogeneity [2]. The WHO classification of GBM provides guidelines for improving its diagnosis and prognosis. There are two main types of GBM—primary and secondary—and the diverging factors that influence the progression of each type include the genotypic status of the tumor, patient age of onset, and history of previous lower-grade diffuse glioma. Primary GBM constitutes around 90% of all cases, it is prevalent in adults aged 55 years and up, and it demonstrates higher malignancy, isocitrate dehydrogenase 1 (IDH1) wild-type status, and poorer clinical outcomes, as median overall survival is around 1.1 years as opposed to 3.8 years for tumors with mutant IDH1 [3]. Studies have further classified GBM into four molecular subclassifications based on information from The Cancer Genome Atlas (TCGA), and these include the classical, mesenchymal, proneural, and neural subtypes [4]. However, an analysis of all these subclassifications is beyond the scope of this review article.

The existing treatment regimen for GBM continues to be the one developed by Stupp and colleagues in 2005 and is currently the gold standard of therapy [5]. Following maximum surgical resection, radiotherapy (RT) and concomitant and adjuvant temozolomide (TMZ) administration are utilized to combat the growth of GBM in adults who are otherwise in good general health and are less than 70 years of age [6]. The dosage of fractionated focal irradiation is set at 2 Gray (Gy) units of ionizing radiation and given 5 days a week for 6 weeks, leading to a total of 60 Gy, and is coupled with continuous daily TMZ administration at 75 mg per square meter (m^2^) of body-surface area per day throughout the entire period of RT. Following RT, TMZ is continued for another six cycles at a dosage of 150 to 200 mg/m^2^ for five days during six 28-day cycles for maintenance. As opposed to solely using surgical resection and RT, the addition of TMZ has increased the median survival by over 2.5 months and the two-year survival rate by over two times while only resulting in grade 3 or 4 hematologic toxic effects in 7 percent of patients [5].

TMZ is an orally administered alkylating agent that has been proven to mitigate the progression of various solid tumors, including GBM. When compared to other similar agents, its small molecular weight, ability to be administered without dietary restrictions, and unique pharmacokinetic properties enable TMZ to efficiently cross the blood-brain barrier, attain high bioavailability, and only induce generally mild side effects [7].

Although TMZ is highly effective in some GBM patients, the O6-methylguanine-DNA methyltransferase (MGMT) gene encodes an enzyme that readily repairs O6-methylguanine (O6-MeG) lesions in the DNA by removing the TMZ-mediated alkyl groups, and MGMT is expressed in around 55% of the patients [8]. Expression of this gene drives a resistant phenotype that often results in treatment failure and worse survival, as shown in clinical trials involving elderly patients in Sweden and Germany [9,10]. Patients who have MGMT promoter methylation fare better, so the presence of epigenetic silencing can be used as a predictive marker for the efficacy of TMZ therapy. A landmark clinical trial conducted by the European Organization for Research and Treatment of Cancer (EORTC) revealed that the current gold standard of therapy involving RT and TMZ increased the median survival of patients with MGMT promoter methylation by 6.4 months, while median survival did not increase a statistically significant amount for patients that did not exhibit MGMT promoter methylation [8].

MGMT promoter methylation is an important predictive marker when evaluating the efficacy of therapy with TMZ; however, a more recent investigation has revealed other mechanisms that can confer therapeutic resistance, such as an increase in drug efflux and activation of cytoprotective autophagy [11]. Autophagy is a cellular homeostatic process that involves the degradation and recycling of intracellular components, such as faulty proteins and organelles, via the use of lysosomes, generating cellular building blocks that can then be recycled in metabolic and biosynthetic pathways. Although present in all cells at basal levels, autophagy is commonly upregulated in the context of cancer to mitigate the effects of stressors such as nutrient deprivation, hypoxia, accumulation of reactive oxygen species (ROS), and cellular damage due to chemotherapy [12].

Dysregulation of this homeostatic process, especially in the context of cancer, gives autophagy the potential to influence invasiveness, motility, chemoresistance, and maintenance of GBM stem cells (GSCs). Unfortunately, even though TMZ can drive the induction of apoptosis through its ability to produce O6-MeG lesions in genomic DNA, TMZ also promotes the induction of cytoprotective autophagy, that, in turn, limits its therapeutic effects. The promotion of autophagy by TMZ is carried out via the ataxia-telangiectasia mutated (ATM) serine-threonine kinase/AMP-activated protein kinase/Unc-51 like autophagy activating kinase-1 (ATM/AMPK/ULK1) axis, which is important in regulating the initiation and maturation steps of autophagy, facilitating the recycling of the damaged intracellular components and avoidance of induction of apoptotic pathways [11].

**Figure 1:**
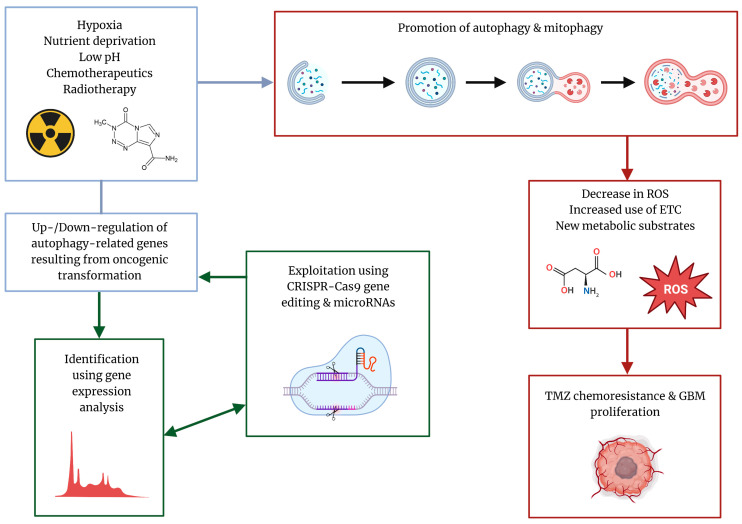
Characteristic stressors in the tumor microenvironment drive selection of tumor phenotypes that are resistant to radiotherapy and chemotherapeutics due to the induction of cytoprotective autophagy and prevention of induction of apoptotic cell death. Identification of autophagy-related genes (ARGs) that are differentially expressed using advanced bioinformatics analysis can enable the selection of novel targets for CRISPR-Cas9 gene editing and miRNA-mediated autophagy inhibition to potentiate TMZ efficacy.

Limitations of the efficacy of current therapeutics in GBM and the presence of processes that confer therapeutic resistance, such as cytoprotective autophagy, have called for the development of novel therapeutic options to combat the progression of this deadly disease and prevent tumor recurrence. Current avenues of investigation include the identification of novel biomarkers to detect levels of autophagy, genome editing with advanced technology such as the clustered regularly interspaced short palindromic repeats (CRISPR)-CRISPR-associated protein 9 (Cas) or CRISPR-Cas9 system to mitigate chemotherapeutic resistance, the use of targeted microRNA (miRNA) to turn off genes that promote autophagy, and employment of the plant-derived bioflavonoids to inhibit autophagy and potentiate the therapeutic action of TMZ in GBM [13,14]. The focus of this review article is to provide a broad overview of the interaction between autophagy and GBM progression and the relationship between TMZ and autophagy, as well as highlight the potential perceived and limitations of current therapeutics being developed (Figure 1).

## 2. Autophagy over the Course of GBM Progression

As a mechanism important for meeting energy requirements and maintaining intracellular homeostasis, autophagy can influence cancer progression. During the initial stages of cancer formation, autophagy can suppress tumorigenesis by limiting several detrimental events such as the accumulation of altered intracellular components, ROS, and damage to the genome. However, in the later stages of cancer, autophagy is activated in a cytoprotective manner that perpetuates cancer progression and can confer resistance to apoptotic cell death even in the hypoxic, nutrient-deprived tumor microenvironment (TME) and following exposure to therapeutics.

Autophagy comes in three forms based on how intracellular components are delivered to the autolysosome for their degradation: macroautophagy, microautophagy, and chaperone-mediated autophagy (CMA) [15]. The most thoroughly investigated form is macroautophagy, which is characterized by the de novo formation of a double-membraned vesicle known as an autophagosome that sequesters cargo such as organelles, protein aggregates, and other soluble proteins. Fusion of the autophagosome with the lysosome results in the formation of an autolysosome for degradation of the cargo via lysosomal acid hydrolases [16]. The overall process of autophagy can be broken down into a series of five main steps that include (i) initiation, (ii) nucleation, (iii) elongation, (iv) maturation, and (v) fusion, and it is regulated by over thirty autophagy-related genes (ATG) [12,14]. On the other hand, microautophagy is not nearly as well characterized, even though it was initially considered the only autophagic pathway in all cells. Rather than employing the formation of an autophagosome, in this case, invaginations in the lysosomal membrane allow for the digestion of small quantities of cytosolic cargo. At the moment, most research on microautophagy has been conducted using the yeast model, so the mechanism of action with respect to mammalian cells is still unclear. Lastly, CMA differentiates itself from macroautophagy and microautophagy in that the cytosolic proteins are targeted selectively for degradation and rather than being engulfed by the formation of a de novo autophagosome membrane or lysosome membrane invaginations, the cargo is translocated across the lysosomal membrane [16]. Macroautophagy, which will be referred to as autophagy (unless specifically stated otherwise) throughout the rest of this review, is the most heavily involved form of autophagy in cancer progression and drug resistance.

The removal of ROS and retrotransposons (the mobile genetic elements known to spread via reverse transcription of RNA intermediates), degradation of abnormal micronuclei and toxic unfolded proteins, and promotion of anti-cancer immunosurveillance all underly the oncosuppressive action of autophagy in initial stages of cancer development [17,18,19,20]. From a mechanistic standpoint, Cordani and colleagues have demonstrated that the wild-type p53, which is an acclaimed tumor suppressor, activates autophagy through a variety of pathways, including the AMPK/mTOR/ULK1 axis [21]. Moreover, the inability to eliminate p62 or sequestosome 1 (SQSTM1) when autophagy is suppressed induces ROS and the DNA damage response [22]. All forms of ROS are very reactive chemicals that are highly genotoxic byproducts of the mitochondrial respiratory chain, and their overproduction can cause oxidative damage to the cellular macromolecules and direct damage to the genomic DNA [17]. Mitochondrial DNA mutations are common in GBM and are often correlated with a malignant phenotype, leading to changes in metabolism, such as compromising oxidative phosphorylation and overproduction of ROS [23]. As such, the selective removal of defective or damaged mitochondria through a specific type of macroautophagy known as mitophagy can aid in mitigating the accumulation of ROS in the initial stages and suppress tumor formation. In addition, autophagy is one of the most primitive examples of an innate immune response, and it is even observed in unicellular organisms [19]. Suppression of autophagy can lead to cell death via necrosis rather than apoptosis, prompting an inflammatory response that can then drive oncogenesis [17,24].

Suppression of autophagy in the initial stages of tumor development may be correlated with oncogenesis; however, it is now clear that autophagy is activated aggressively as a cytoprotective mechanism in the advanced stages of tumor progression, as it is constitutively upregulated in many cancer types, including GBM [12,25,26,27,28]. The recycling of the damaged and dysfunctional intracellular components via autophagy can enable cancer cells to survive in the face of hypoxia, nutrient deprivation, and exposure to toxic therapeutics, maintain the GSCs and promote metastatic potential and invasiveness [29,30,31,32].

The upregulation of autophagy in tumor cells in response to hypoxia is highly evident by an increased number of autophagosomes that are localized in the hypoxic areas of the TME [18]. In addition, a relationship has been found between the upregulation of a downstream regulator of hypoxia-inducible factor 1-alpha (HIF1-α) known as the Bcl-2/adenovirus E1B 19 kDa interacting protein 3 (BNIP3) and increased turnover of p62, indicating that hypoxia increases autophagic flux (amount of autophagic degradation activity) in GBM [33]. Similarly, the upregulation of autophagy plays a key role in maintaining optimal bioenergetics even in an environment characterized by nutrient depletion. Autophagy can degrade organelle membranes and lipid droplets to generate lipids, proteins to generate amino acids, and sugars through complex carbohydrate degradation to create a huge pool of building blocks that can then be fed into the tricarboxylic acid (TCA) cycle to sustain mitochondrial metabolism [18]. Specifically, a cluster of differentiation 133 (CD133), which is commonly used as a molecular marker for cancer stem cells, has been shown to associate with Atg5, Beclin 1, and lysosomes, and CD133-positive cells exhibit increased levels of autophagy and the ability to adapt to the conditions of nutrient deprivation [34].

Moreover, tumor cells often modify mitochondrial dynamics to meet cellular energy needs, either utilizing fusion or fission of mitochondria to increase utilization of the electron transport chain or reduce oxidative capacity, respectively [35]. Mitochondrial fission is common in GBM, and the facilitation of a high turnover of mitochondria via mitophagy plays a critical role in the maintenance of the cancer stem cell phenotype [36,37]. Capparelli and colleagues have proposed a mechanism for tumor metabolism that consists of two compartments: (a) oxidative stress in the stroma upregulates autophagy, leading to the production of high-energy mitochondrial fuels such as ketone bodies and free fatty acids that can then be used for oxidative phosphorylation and (b) oxidative mitochondrial metabolism in other cancer cells [38].

Autophagy plays a dual role in tumor progression that has changed the way it has been characterized over time, but at this moment, there is more evidence showing that autophagy potentiates cancer progression rather than its suppression, indicating that autophagy is detrimental in progressive cancers. As such, targeting autophagy is a potential therapeutic option to mitigate GBM progression, invasion, and recurrence.

## 3. TMZ Can Function as an Autophagy Promoter in GBM

One of the first Phase I clinical trials of TMZ was conducted in 1992 on patients with advanced cancer refractory to the standard of care at the time and included those diagnosed with melanoma, high-grade gliomas, ovarian cancer, and lymphoma [39]. Unlike mitozolomide, a precursor also assessed in Phase I clinical trials, TMZ was able to provide high oral bioavailability without causing severe and unpredictable myelosuppression [40]. By the end of 1993, Phase II trials had shown success in patients with high-grade astrocytomas, and in 2005, Stupp and colleagues demonstrated that a concomitant and adjuvant schedule of TMZ administration with radiotherapy could greatly improve the outlook for patients with GBM, which was a groundbreaking treatment outcome as many trials until that point in time had only tested adjuvant use of chemotherapeutics [5].

As a part of the imidazotetrazine family of chemotherapeutics, the molecular weight of TMZ is 194 Daltons (Da), and its lipophilic properties facilitate oral administration and provide high bioavailability. Administered as a prodrug, when TMZ comes into contact with the slightly basic physiological pH of the blood and tissues, it is converted into 5-(3-dimethyl-1-triazenyl) imidazole-4-carboxmide, or MTIC, which is the active form of the drug [41]. Further hydrolysis of MTIC yields 5-aminoimidazole-4-carboamide (AIC) and the highly reactive methyldiazonium cation (CH_3_N_2_^+^) that then goes on to methylate guanine residues in the genomic DNA [42]. DNA alkylations or lesions are commonly produced in three separate locations—the N7 position of guanine, the N3 position of adenine, and the O6 position of guanine (O6-MeG)—overall leading to the formation of O6-MeG being the main alkylations and drivers for induction of apoptosis [43]. An important property to note here is that the mechanism of action of TMZ does not involve the cross-linking of DNA, as is observed in treatment with nitrosoureas, platinum compounds, and procarbazine, which explains its lower adverse effects on the patients [7].

TMZ has demonstrated an ability to upregulate autophagy in GBM both in vitro and in vivo [41,44]. Suppressing the phosphoinositide 3-kinase (PI3K) and protein kinase B (also known as Akt) axis while upregulating the mitogen-activated protein kinase (MAPK) pathway can lead to enhancement of autophagy activation following TMZ administration [45]. An increase in activation of autophagy has been tracked on the molecular levels, showing statistically significant changes in the microtubule-associated protein 1 light chain 3 isoform B (colloquially called LC3B), lysosome-associated membrane protein 1 (LAMP1), and LAMP2A following treatment with TMZ, indicating an upregulation of cytoprotective autophagy in patient samples. In addition, a histological analysis of the recurrent tumors in patients following TMZ treatment revealed that the new tumors exhibited increased levels of anaplasia and autophagy, upsetting the treatment outcomes [44]. Phase I and Phase II clinical trials are being conducted using an autophagy inhibitor drug in combination with TMZ for targeting autophagy in GBM (Table 1).

The relationship between the regulation of bioenergetics and the induction of autophagy via the action of TMZ is also important in the context of GBM. Studies of a group have shown a direct relationship between autophagy induction and adenosine triphosphate (ATP) production in response to TMZ treatment [52]. The conversion of LC3B into LC3B-II and LC3B-I fragments is an indicator of autophagic flux, which is a measure of autophagic degradation activity, as mentioned earlier. Tracking the levels of LC3B-II, a phosphatidylethanolamine (PE)-conjugated form of LC3B-I, and downstream effectors of mTOR, they found that TMZ increased levels of autophagy even only three days after its administration and that autophagic flux correlated with changes in ATP levels. To confirm the relationship between autophagy and ATP production, they inhibited autophagy utilizing 3-methyladenine (3-MA), an early-stage autophagy inhibitor, and a short hairpin RNA (shRNA) that targeted Beclin 1, an integral protein in the nucleation phase of autophagy progression. Both 3-MA and shRNA suppressed the ATP surge caused by TMZ and led to higher levels of micronucleation and subsequent cell death in multiple GBM cell lines [52].

Production of O6-MeG resulting from the action of TMZ has been related to the induction of autophagy, senescence, and apoptosis in a time-dependent manner [53]. Although senescence has been thought of as a favorable end-result of chemotherapeutic action in the past, increasing evidence has begun to show that the chemokines, cytokines, growth factors, extracellular vesicles, and other soluble factors that are released by the senescent cells as part of the senescent-associated secretory phenotype (SASP) can lead to a pro-inflammatory response that promotes tumorigenesis [54]. Multiple groups have found that treatment with TMZ leads to an activation of autophagy that promotes senescence and avoidance of induction of apoptotic pathways, and the induced senescence subsequently favors tumor recurrence [53,55,56]. Attainment of senescence following a highly proliferative state can be explained by the activation of autophagy as a mechanism that involves rapid protein remodeling to transition between the states [56]. On the one hand, Young and colleagues found that overexpression of the pro-autophagic gene ULK3 induced both autophagy and senescence. In contrast, Knizhik and colleagues found that inhibition of autophagy using the early-stage autophagy inhibitor 3-MA led to the complete abolition of senescence following TMZ treatment [53,56].

The role of TMZ-induced autophagy in the regulation of senescence is further complicated by the presence of GSCs. Reduced senescence was observed in GBM cells that were cultured as multicellular spheroids, with a growth rate significantly less affected by TMZ treatment when compared with the human U87MG cell line [57]. In addition, the knockdown of Beclin 1 and ATG5 leads to down regulation of autophagy, which in turn results in the enhancement in expression of GSC markers, particularly CD133/prominin-1, which is a transmembrane glycoprotein. Moreover, the knockdown of Beclin 1 led to cell lines that were more likely to develop spheres, while ATG5 knockdown cell lines exhibited cells that were smaller in comparison to controls and did not form spheres, showing that specific proteins involved in autophagy progression might differentially regulate stemness [58]. Autophagy can also act in a cytoprotective manner in GSCs, as activation of the AMPK/ULK1 pathway by the protein death-ligand 1 (PD-L1) containing exosomes derived from GSCs leads to induction of autophagy and TMZ resistance that can subsequently be reversed by 3-MA treatment [59]. As a result, the relationship between TMZ-induced autophagy, stemness, and differentiation is complex and requires further investigation.

To address the roles of cytoprotective autophagy, induction of senescence, modulation of bioenergetics, and maintenance of stemness that results from TMZ treatment in GBM, various additional novel therapeutic strategies have been developed to use in combination with the current gold standard to improve patient outlook. Examples include the application of advanced bioinformatics analysis for identifying autophagy biomarkers, exploration of gene editing utilizing CRISPR-Cas9 technology, and the use of other technologies such as miRNAs and viral vectors to modulate gene expression in GBM, all of which will be subsequently described in greater depth.

## 4. Bioinformatics Analysis for Identification of Biomarkers of Autophagy in GBM

Development of genomic databases, novel quantitative proteomic analysis techniques and increased investigation in the field of autophagy regulation in GBM in response to radio-resistance and chemo-resistance has yielded unprecedented opportunities to select genomic and proteomic targets that have been shown to be dysregulated in large populations of patients (Table 2).

A statistical analysis of large genomic and proteomic datasets has led to the creation of various nomograms (diagrams representing relations among various key parameters) and protein-protein interaction (PPI) networks that can be used to determine how variations in specific autophagy-related genes (ARGs) can affect prognosis and drive individualized treatments [60,61,62]. Utilizing data from the Cancer Genome Atlas (TCGA), Kondapuram and Coumar have conducted a pan-cancer gene expression analysis, determining which ARGs are commonly upregulated or down regulated across 21 different cancers [60]. They have found that the most frequently upregulated across all cancers are cyclin-dependent kinase inhibitor 2A (CDKI2A) and baculoviral inhibitor of apoptosis repeat-containing 5 (BIRC5), both of which have been shown to be dysregulated in GBM. Specifically, CDKI2A is deleted in 57.8% of GBM patients, leading to dysregulation of the p53 pathway, while BIRC5 interacts with nuclear factor-kappaB (NF-κB) to decrease GBM sensitivity to epidermal growth factor receptor (EGFR) tyrosine kinase inhibitors [55,63]. Focusing on the ARG expression analysis on GBM yielded two genes that have significant effects on overall survival (OS) in GBM: integrin subunit beta 1 (ITGB1) and BIRC5, as mentioned above. The ITGB1 gene was found to map to the phagosome pathway, specifically the PI3K/Akt/mTOR axis, and be involved in autophagosome formation. Using this information, three drugs were identified to target commonly dysregulated ARGs and suggested for GBM therapy: panabinostat, vorinostat, and abexinostat [60].

**Table 2:** Key autophagy-related genes (ARGs) involved in GBM progression and respective models.

Identified ARGs	Role in Autophagy and GBM Progression	Database(s) Used	AUC Values	References
CDK12A	Dysregulates p53 tumor suppressor pathway	TCGA	NA	[55,60,63]
BIRC5	Reduces sensitivity to EGFR tyrosine kinase inhibitors			
ITGB1	Involved in autophagosome formation			
ITGA3	Promotes FAK/PI3K/Akt pathway; interacts with ITGB1 to act as cell surface adhesion molecule	TCGA (Identification), CGGA (Verification)	0.76 (0.5 yr), 0.72 (1 yr), 0.69 (2 yr)	[61,64,65,66,67]
NRG1	Promotes autophagy via ERK1/2 activation			
MAP1LC3A	Biomarker of autophagy progression			
DIRAS3	Promotes autophagy via EGFR/Akt axis	TCGA (Identification) REMBRANDT and Gravendeel (Verification)	0.627 (1 yr), 0.733 (3 yr), 0.64 (5 yr)	[62,68,69]
LGALS8	mTOR inhibitor			
STAM	ULK1 stabilization and JNK1 upregulation			
UBC	Inhibits autophagosome formation	TCGA (Identification and verification)	0.811 (Combined models)	[70,71,72,73,74,75,76]
VHL	Inhibits autophagy by decreasing HIF-1α stability			
KCTD7	Dysfunction correlated with autophagy defects			
FBXL19	Induces chemotherapy resistance via miR-203 sponging			
RNF7	Indirectly activates autophagy via RNF7/CARD14/NF-κB axis			

CARD14, caspase recruitment domain family member 14; NA, not available.

Utilizing the TCGA and univariate and multivariate Cox proportional hazards regression, other research groups found additional differentially expressed ARGs that could function as potential targets for therapy in GBM. Wang and colleagues found cohorts of GBM patients with lower expression of integrin subunit alpha 3 (ITGA3), neuregulin 1 (NRG1), and microtubule-associated protein 1 light chain 3 alpha (MAP1LC3A) to have a significantly better prognosis. ITGA3 silencing has been shown to correlate with inhibition of the focal adhesion kinase (FAK)/PI3K/Akt pathway, and ITGA3 also joins with ITGB1 to function as a cell surface adhesion molecule, leading to worse prognosis in other cancers such as intrahepatic cholangiocarcinoma [64,65]. NRG1 is a cytokine that promotes GBM survival through activation of the extracellular signal-regulated protein kinases 1 and 2 (ERK1/2), which have been shown to upregulate autophagy via Beclin 1 [14,66]. Lastly, MAP1LC3A, also known as LC3A, is a well-known biomarker of autophagy progression, as changes in LC3A-I to LC3A-II ratios can quantify autophagy flux [67]. Utilizing these three targets, a risk score model was created where patients were divided into low-risk and high-risk groups using the median risk score as a cutoff [61]. Patients that were in the high-risk group had a one-year OS of 39.5%, while those in the low-risk group had an OS of 73.4%, showing that these might be viable targets for therapy. The nomogram was verified using data from the Chinese Glioma Genome Atlas (CGGA) and achieved AUC (area under the receiver operating characteristic curve) values of 0.76, 0.72, and 0.69 for 0.5-, 1-, and 2-year OS rates, respectively.

Another nomogram that achieved relatively high AUC values for 1-, 3-, and 5-year OS for GBM was developed using the TCGA, REMBRANDT (an acronym for: REpository for Molecular BRAin Neoplasia DaTa), and Gravendeel datasets. The least absolute shrinkage and selection operator (LASSO) and multivariate Cox regression yielded four ARGs that were identified as either risk factors or protective factors. With a hazard ratio (HR) greater than one, Di-Ras (DIRAS) family GTPase 3 (DIRAS3) and galectin-8 (LGALS8) are known to promote autophagy either through the EGFR/Akt axis or through mTOR inhibition, respectively [62]. On the other hand, mitogen-activated protein kinase 8 (MAPK8)/c-Jun N-terminal kinase 1 (JNK1) and signal transducing adaptor molecule (STAM) had an HR of less than 1, indicating that they were protective factors in GBM tumorigenesis. Interestingly, other groups have found STAM upregulation to be related to ULK1 stabilization and JNK1 upregulation to lead to activation of Beclin 1, indicating that both ARGs work in promoting autophagy [68,69]. Verifying the TCGA-derived cohort of 525 GBM patients using the REMBRANDT and Gravendeel databases, they achieved AUC values for 1-, 3-, and 5-year OS of 0.583, 0.824, and 0.799, respectively, for the Gravendeel dataset and 0.627, 0.733, and 0.64, respectively, for the REMBRANDT dataset [62].

Additional genomic analysis has focused on the relationship among ARG expression, immune infiltration, and alternative splicing (AS) [70,77]. High expression levels of NADH (nicotinamide adenine dinucleotide + hydrogen)-ubiquinone oxidoreductase subunit B9 (NDUFB9), glyceraldehyde-3-phosphate dehydrogenase (GAPDH), cyclin-dependent kinase inhibitor 1B (CDKN1B), charged multivesicular body protein 6 (CHMP6), and EGFR correlated significantly with increased infiltration of CD8+ T cells, while CDKN1B expression was also correlated positively with macrophage level [77]. The identification of the relationships between commonly upregulated ARGs and immune response within the TME can potentially aid in the selection of novel targets, as autophagy has been shown to confer resistance to anti-cancer immunity [78]. Moreover, analysis of AS in 134 GBM patients utilizing the Kyoto Encyclopedia of Genes and Genomes (KEGG) revealed that autophagy is the most enriched process of prognostic AS in GBM. Spliced core genes identified following univariate and multivariate Cox regression of the TCGA data included ubiquitin C (UBC), von Hippel-Lindau tumor suppressor (VHL), potassium channel tetramerization domain containing 7 (KCTD7), f-box and leucine-rich repeat protein 19 (FBXL19), ring finger protein 7 (RNF7), and ubiquitin-conjugating enzyme E2 N (UBE2N). Although there are other groups that have identified specific examples of AS leading to impairment of autophagy in prostate cancer and nervous tissue, Xie and colleagues have noticed that the relationship between AS and autophagy is still relatively unclear and will require further investigation [70,79,80].

Other avenues of investigation that focus on the identification of novel biomarkers include proteomic analysis and knockdown of long non-coding RNAs (lncRNAs) that promote radiotherapy and chemotherapy resistance in GBM. lncRNAs belong to a large and diverse class of RNA molecules with a length of more than 200 nucleotides that do not encode proteins but exert their functions either by binding to DNA or RNA in a sequence-specific manner or by binding to proteins. Utilizing tandem mass tag (TMT) quantitative proteomic analysis, syndecan 1 (SDC1) and transglutaminase 2 (TGM2) were shown to interact with ectopic P-granules 5 autophagy tethering factor (EPG5) to facilitate fusion of the autophagosome with the lysosome, driving autophagy and radiotherapy resistance in GBM cells [81]. Moreover, expression levels of the lncRNA CRNDE (colorectal neoplasia differentially expressed) were correlated with poor prognosis in 58 cases of GBM tissue specimens, and knockdown of this lncRNA led to activation of the PI3K/Akt/mTOR pathway, inhibition of autophagy, and increase in TMZ sensitivity in GBM [82].

Biomedical informatics, growing genomic databases, novel methods for proteomic analysis, and identification of key miRNAs and lncRNAs in GBM provide some unparalleled opportunities for selecting targets that can curb the progression of GBM. However, certain limitations continue to exist for the development of nomograms that may potentially be used to identify key ARGs and pharmaceutical interventions that target these ARGs. Much of the data procured from various datasets, such as the TCGA, REMBRANDT, Gravendeel, and KEGG, are of limited sample size and may not contain valuable information such as the extent of surgical resection for development of prognostic weightings and few experimentally verified connections between modulation of gene expression and involvement in specific intracellular pathways [62,77]. These inconsistencies can potentially explain the discrepancies among the studies conducted and will need to be addressed in the future to create actionable data through additional investigation and a more thorough understanding of the PPI networks that guide a key cellular process like autophagy. In the meantime, many advances in genetic technologies, such as gene editing utilizing CRISPR-Cas9 technology and mapping important miRNAs and lncRNAs, can supplement genome-wide analysis to detect and target novel biomarkers of cytoprotective autophagy for inhibition of GBM growth.

## 5. Gene Editing Technology for Targeting Cytoprotective Autophagy in GBM

The discovery of the double-helical structure of DNA spurred the development of a novel field of investigation: the creation of a mechanism for targeted genome editing. First came oligonucleotides, which could induce site-specific chromosome modification when coupled to chemical cleavage or cross-linking reagents. These were then replaced by self-splicing introns, which consisted of intron-encoded nucleases that could affect site-specific DNA cleavage. Although both methods showed some promising applications, they were not consistently reproducible. The creation of modular DNA recognition proteins utilizing the zinc ion-regulated small protein motifs and the nuclease domain of the Fok1 restriction nuclease, leading to what would be called zinc finger nucleases (ZFNs), represented a major advancement in the field of gene editing; however, validation and protein design proved to be barriers to the widespread use of ZFNs [83]. Similarly, although transcription activator-like (TAL) effector nucleases (TALENs) were even more efficient than ZFNs to produce, challenges with protein design and synthesis persisted [84].

**Figure 2:**
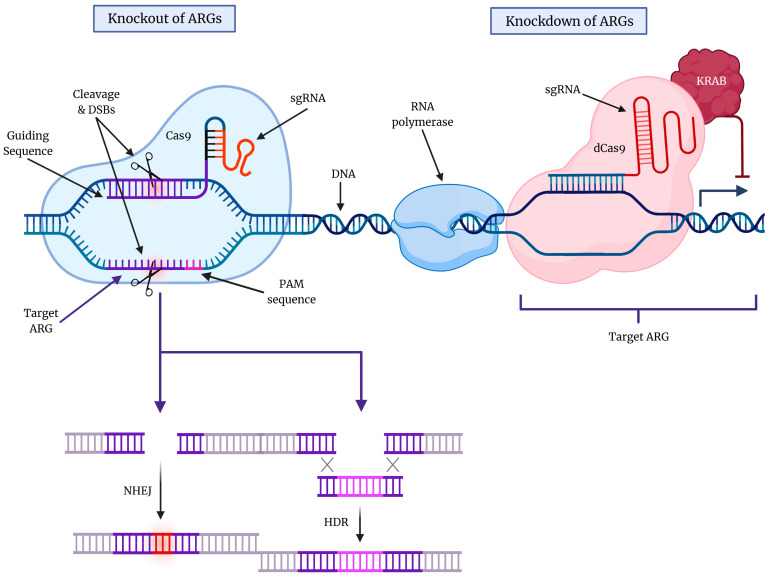
An overview of the CRISPR-Cas9 gene editing technology. The ever-evolving CRISPR-Cas9 gene editing technology has enabled the knockout (KO) and knockdown (KD) of specific genes that are known to promote cytoprotective autophagy in cancers. The protospacer adjacent motif (PAM) sequence and sgRNA are essential for DNA target recognition. The double-strand breaks (DSBs) that result from CRISPR-Cas9 action can lead to either non-homologous end joining (NHEJ) or homology-directed repair (HDR) in the DNA molecule. KO typically involves NHEJ, which produces indels that lead to frameshift mutations, while HDR is another mechanism of DNA repair that involves the insertion of a specific DNA template utilizing homologous regions to rejoin cleaved DNA (homologous regions are shown by X). On the other hand, the use of catalytically deactivated Cas9 (dCas9) enables the reversible KD of ARGs, blocking transcriptional elongation, RNA polymerase binding, and/or transcription factor binding.

The DNA sequence of CRISPR was first characterized in 1987 by Ishino and colleagues when examining the genome of *Escherichia coli* [85]. Subsequent investigation revealed the importance of CRISPR-associated (Cas) genes that encode proteins with nuclease and helicase domains, mature CRISPR RNAs (crRNAs) that can guide the formation of complex Cas proteins, and trans-activating crRNA (tracrRNA) for crRNA maturation. Moreover, identification of the HNH (His-Asn-Hos) and RuvC domains of Cas9 has aided in the creation of nickases that potentially have reduced off-target effects, while the tracrRNA:crRNA duplex is now replaced by a single guide RNA (sgRNA), which has a 20-nucleotide sequence that can be easily modified to enable editing at almost any point in the DNA molecule [84]. Major applications of CRISPR-Cas9 technology include the reproduction of tumor-associated chromosomal translocations and the creation of more accurate disease models, systemic analyses of gene function, and correction of genetic mutations. Importantly, some recent advances in CRISPR-Cas9 technology include the refinement of base-editing technology, catalytically deactivated Cas9 to block transcriptional elongation, RNA polymerase binding, transcription factor binding, and use as a high-throughput assay for functional screening of large sets of genes [83,84] (Figure 2).

### 5.1. CRISPR-Cas9 Technology for Targeting Molecular Components of Autophagy

As an effective method of identifying the action of individual genes, CRISPR-Cas9 technology has been employed for creating knockouts (KOs) of various ARGs that regulate canonical autophagy pathways and organelle-specific forms of autophagy [86,87]. ARGs involved in canonical autophagy pathways that have undergone KO include ATG7, which is integral for autophagosome formation, leading to a blockage of basal and starvation-induced autophagic flux and substantially greater cell death in the immortalized human embryonic kidney cell line HEK293T [86,88]. Other groups have knocked out ULK1, leading to decreased tumor necrosis factor (TNF) secretion and selective autophagy, and ATG5 to determine alternative pathways for the induction of autophagy [89,90].

Determining the role of key ARGs in organelle-specific autophagy, such as mitophagy and ER-phagy, has led to fervent investigation and elucidation of novel pathways. The ATG8 protein family consists of two subdivisions: LC3 and gamma-aminobutyric acid (GABA) type A receptor-associated protein (GABARAP). Known to be an integral component in autophagosome expansion and closure, as well as in sequestration of selective cargo, their relationship with the phosphatase and tensin homolog (PTEN) induced kinase 1 (PINK1)/Parkinson disease 2 (PARK2)-dependent mitophagy was explored. Utilizing CRISPR-Cas9, KO of LC3 and GABARAP subfamilies and an additional six ATG8 family proteins in HeLa cells led to the failure of autophagosome-lysosome fusion but did not lead to complete prevention of autophagosome closure or selective sequestration of mitochondria. Additional interrogation revealed that GABARAPs recruited the pleckstrin homology and RUN domain containing M1 (PLEKHM1) for autophagosome-lysosome fusion, providing novel insight into the mechanics of PINK1/PARK2-dependent mitophagy [91]. Moreover, Wang and colleagues found that even in the ATG7 KO chronic myelogenous leukemia K562 cells, mitophagy continued to be activated due to the action of the Ras-related protein Rab-9A (RAB9A) and that KO of RAB9A inhibited mitophagy, increased ROS and apoptosis, and reduced repair of DNA damage [92]. Other studies utilizing CRISPR-Cas9 technology have shown the utility of the adenine nucleotide translocator (ANT) complex in the inhibition of the mitochondrial import inner membrane translocase unit Tim23 (TIM23) complex and stabilization of PINK1 and of hexokinase 2 (HK2) in the assembly of high molecular weight PINK1 complex, resulting in accumulation of phosphorylated ubiquitin in response to mitochondrial damage [93,94]. Within the context of ER-phagy, knockdown (KD) of mitochondrial genes integral to the function of oxidative phosphorylation (OXPHOS) led to the repression of ER-phagy, revealing novel connections between different organelle-specific forms of autophagy [87].

### 5.2. CRISPR-Cas9 Technology for Targeting GBM Growth and Recurrence

Genome-wide screening yielded novel regulators of autophagy machinery that could potentially be targeted by therapeutics (Table 3). Upstream of autophagosome formation, the KO of phosphoribosyl formylglycinamidine synthase (PFAS), which is a de novo purine synthesis enzyme, leads to activation of autophagy, adding complexity to autophagy activation following intracellular purine starvation via the tuberous sclerosis 2 (TSC2)/Ras homolog (RHEB)/mTORC1 axis. However, even under purine starvation conditions, the KO of TSC led to the inhibition of autophagy [95]. This particular axis has been exploited in the context of GBM, with KO of TSC2 leading to an increase in sensitivity to photodynamic therapy [96]. Downstream of autophagosome formation, transmembrane protein 41B (TMEM41B), which is an ER-localized lipid scramblase, is identified to be integral to autophagosome formation in K562, HEK293, and neuroglioma H4 cells and KO of TMEM41B leads to inhibition of autophagy [97,98,99]. Moreover, LC3B tagging with a tandem of green fluorescent protein (GFP) and mCherry fluorescent protein (in short, tf-LC3B) revealed that ubiquitin-like modifier activating enzyme 6 (UBA6) and BIRC6 negatively regulated LC3B upstream via ubiquitination, indirectly inhibiting autophagy [100].

The application of CRISPR-Cas9 technology to target GBM growth has primarily taken place in four areas: mitigation of GSC proliferation, modulation of epigenetics, animal modeling and organoid development, and immunotherapy. A key target identified for CRISPR-Cas9 mediated KO is an enhancer that is located between the promoters of the marker of proliferation Ki67 (MKI67) and MGMT genes. By preventing epigenetic regulation of the enhancer region in cell lines with high MGMT expression, TMZ sensitivity was restored, impairing the proliferation of GBM12 cells [101].

**Table 3:** Genes edited to inhibit autophagy and counteract GBM progression.

**General Macroautophagy**			
**KO of Gene(s)**	**Cell Line/Type**	**Therapeutic Outcome and Mechanism**	**References**
ATG7, ULK1, and ATG5	HEK293T	Inhibition of autophagy and decreased TNF secretion	[88,89,90]
TSC2	HEK293T and LN18	Inhibition of autophagy even in purine starvation conditions; increased sensitivity to photodynamic therapy	[95,96]
TMEM14B	H4	Inhibition of late-stage autophagy by preventing formation of mature autophagosomes	[97,98]
BIRC6	H4	Inhibition of autophagy via upstream ubiquitination of LC3B	[100]
STAT3	GSCs	Inhibition of GBM proliferation and autophagy via association of Bcl-2 and Beclin 1	[99,102,103,104]
**Mitophagy**			
**KO of Gene(s)**	**Cell Line**	**Therapeutic Outcome and Mechanism**	**References**
GABARAP and 6 other ATG8 genes	HeLa	Inhibition of autophagosome-lysosome fusion due to impaired recruitment of PLEKHM1	[91]
ATG7 and RAB9A	K562	Prevented initiation of alternative mitophagy, leading to ROS accumulation and DNA damage	[92]
HK2, SEC22B, and WIPI2	HeLa	Demonstrated greatest inhibition of mitophagy in screen	[94]
**ER-phagy**			
**KD of Gene(s)**	**Cell Line**	**Therapeutic Outcome/Mechanism**	**References**
NDUFB4 and NDUFB2	HCT116	KD of components required for OXPHOS impaired ER-phagy independent of AMPK signaling	[105]
**Other Applications**			
**KO of Gene(s)**	**Cell Line/Type**	**Therapeutic Outcome/Mechanism**	**References**
MKI67	GBM12	Decrease in MGMT expression and restoration of TMZ sensitivity	[101]
PKMYT1	GSCs	Essential for mitosis and GSC proliferation by inhibition of cyclin B-CDK1 activity	[106]
DGK	CAR-T cells	Significant regression of U87MG xenografted model via activation of ERK	[107]
PD-1, TRAC, and B2M	CAR-T cells	Enhanced survival of GBM xenografts via increased production of proinflammatory cytokines	[108]

Identification of the genes that are differentially upregulated in GSCs could provide a novel target for therapeutic interventions to prevent the recurrence of GBM. One exciting result from the CRISPR-Cas9 gene editing was that the KO of the signal transducer and activator of transcription 3 (STAT3) led to significant inhibition of GBM proliferation, preferentially targeting GSCs when the gene editing technology was delivered via intracranial injection [102]. STAT3 has already been shown to be an upstream inhibitor of autophagy through multiple axes, the most well-established being the upregulation of hypoxia-inducible factor-1 alpha (HIF-1α), followed by an increase in the association of Bcl-2 and Beclin 1, resulting in down-regulation of autophagy [103,104]. Differential effects of autophagy upregulation and down regulation in GSCs as opposed to other GBM cells can potentially explain how KO of STAT3 may be beneficial in the context of GSCs. Another gene, which was identified through the CRISPR-Cas9 screening to be expressed in a manner that implied an ‘addiction’ in GSCs when compared to neural stem cells (NSCs), was WEE1 (a protein kinase for regulating the G2 checkpoint in the cell cycle in response to DNA damage)-like kinase (PKMYT1) that was essential for mitosis. In neural stem cells (NSCs), redundancy with WEE1 prevents inhibition of mitosis following KO of PKMYT1; however, overexpression of EGFR and Akt1 overcomes redundancy [106]. WEE1 has also been recently analyzed with respect to autophagy in GBM, and the lncRNA LINC00470 has been found to competitively bind to miR-580-3p in the presence of WEE1, leading to autophagy inhibition via activation of the PI3K/Akt/mTOR pathway [109]. Stem cell-specific models have also been developed using the CRISPR-Cas9 technology. Combination of the RCAS-TVA (replication-competent avian sarcoma-leukosis virus long-terminal repeat with splice acceptor (RCAS) vectors targeting the tumor virus A (TVA) receptor) method with the CRISPR-Cas9 gene editing technology enabled KO of p53, CDKN2A, and PTEN in NSCs, resulting in the formation of high-grade glioma [110]. Essentially, the CRISPR-Cas9 gene editing technology is an efficient method to screen many genes to determine what differentials may exist between GSCs and other GBM cells, as well as create representative models of GSCs to understand the intricacies of tumorigenesis.

Modeling GBM in vivo and through organoids has been another area of application of CRISPR-Cas9 gene editing technology to better understand the pathogenesis of GBM, investigate loss-of-function (LOF) mutations of tumor suppressors, engineer oncogene constructs, and improve target identification [111,112]. In the case of the animal model, Zuckermann and colleagues were able to induce the formation of GBM through the KO of p53, PTEN, and neurofibromatosis type 1 (NF1) [113]. Using electroporation into the ventricular zone, about 6-14 weeks after the delivery, they observed histological features that matched those of GBM [113]. The development of organoids is another promising alternative to the animal model, as they can accurately resemble organ histology and physiology. An example of one such model is a neoplastic cerebral organoid, colloquially called neoCOR and created by Bian and colleagues, which can be perturbed by the KO of CDKN2A, CDKN2B, NF1, PTEN, and p53 as well as amplification of EGFR utilizing CRISPR-Cas9 technology [114]. The ability to initiate tumorigenesis through mutation of only a small population of cells within the organoid makes it highly possible to more accurately mirror GBM proliferation in vivo [114]. Another instance of the use of organoids to model GBM pathogenesis was employed by Ogawa and colleagues, who used CRISPR-Cas9 gene editing technology to KO p53 and amplify oncogenic HRas^G12V^ [115]. Akin to Bian and colleagues, the CRISPR-Cas9 machinery, in this case, was only delivered to a small set of cells, and these cells rapidly became invasive, destroying surrounding structures and overwhelming the entire organoid [115]. These organoid models may well be used to elucidate how key autophagy genes impact tumorigenesis and proliferation in the initial stages of GBM development as well as to identify novel targets for therapies.

Targeted immunotherapy in GBM has been another area in which CRISPR-Cas9 gene editing technology has been employed to reduce sensitivity to immunosuppression and improve anti-tumor T cell activity. Autophagy can increase resistance to anti-cancer immunity, so the identification of novel targets for the potentiation of immunotherapy may mitigate the effects of cytoprotective autophagy [78]. Chimeric antigen receptor (CAR)-T cells were shown to be less sensitive to immunosuppressive factors such as transforming growth factor beta (TGFβ) and prostaglandin E2 (PGE2) following CRISPR-Cas9 mediated KO of diacylglycerol kinase (DGK) [107]. The ultimate result was the significant regression of the U87MG EGFRvIII-positive tumor and proliferation of CAR-T cells through the activation of extracellular signal-related kinase (ERK) signaling in a xenografted mouse model [107]. Targeting programmed cell death protein-1 (PD-1) with CRISPR-Cas9 gene editing technology also led to the reduction of CAR-T cell alloreactivity (a robust T cell reactivity against allelic variants of the major histocompatibility complex (MHC) molecules), the prevention of apoptosis, and a decrease in sensitivity to immunosuppression [116]. Moreover, another group utilized combined KO of PD-1, endogenous T-cell receptor (TCR) alpha chain (TRAC), and beta-2 microglobulin (B2M) to enhance survival in a GBM xenografted mouse model [117]. These studies elegantly show the promise of the use of CRISPR-Cas9 gene editing technology in GBM immunotherapy to potentially complement autophagy inhibition to counteract the progression of GBM growth.

Now going beyond its ten-year mark since the publication of the landmark paper in *Science* first describing how CRISPR-Cas9 technology can be used for precise gene editing, much investigation has taken place with respect to the identification of the function of various genes related to the regulation of autophagy and the elucidation of applications to GBM characterization and therapy [118]. However, the use of CRISPR-Cas9 technology for the modification of ARGs in GBM is still incredibly limited [13]. Leveraging CRISPR-Cas9 modified organoid models and CAR-T cells with decreased sensitivity to immunosuppressive factors, a TME and GBM model conducive to functional analysis of ARGs may reveal novel targets that will potentiate the therapeutic action of TMZ intended specifically for induction of apoptosis.

## 6. miRNAs and Inhibition of Autophagy for Potentiation of TMZ Efficacy in GBM

**Figure 3:**
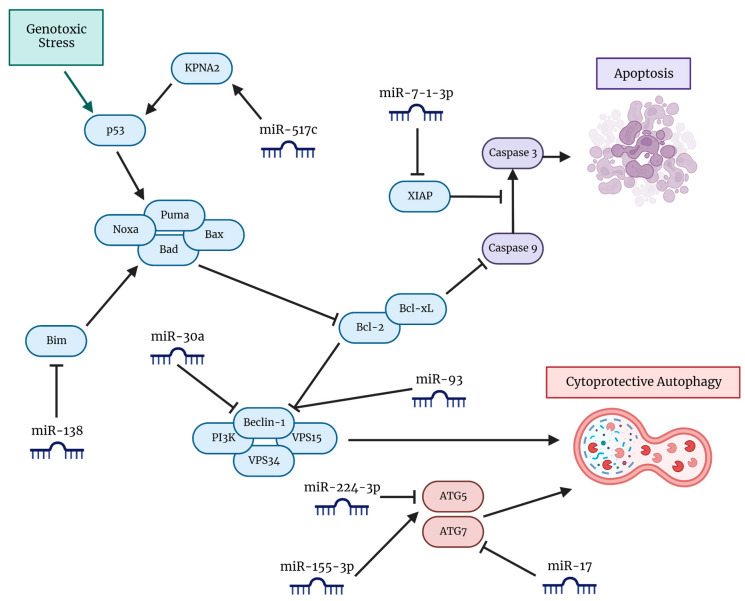
A visual representation of pathways with involvement of miRNAs in the induction of cytoprotective autophagy and inhibition of apoptosis in GBM cells. Various miRNAs directly interact with the pathways involved in autophagy progression to either promote or inhibit autophagy. Key pathways include those involving proteins that are members of the Bcl-2 family, such as Bim, Bad, Bax, Bcl-2, Bcl-xL, Beclin 1, and the tumor suppressor p53. Modification of expression of these miRNAs has led to increased TMZ chemosensitivity and synergism in combatting GBM.

Following their discovery in 1993, the role of non-coding RNAs (ncRNAs) in the regulation of gene expression has opened new doors for the diagnosis of disease, prediction of patient prognosis, and the manipulation of their activity for therapeutic intervention [119]. Small ncRNAs termed miRNAs were first described in the context of cancer in 2002, with certain miRNAs being differentially downregulated in B-cell chronic lymphocyte leukemia (B-CLL) [120]. Since then, an intensive investigation has led to the characterization of key miRNAs and their action within a variety of pathways integral to cancer progression, radio-resistance and chemo-resistance [121] (Figure 3). Elucidating and exploiting miRNAs that can inhibit autophagy to synergistically counteract the progression of GBM growth in concert with TMZ treatment is currently an active area of investigation.

Frequently, miRNA targets are chosen following quantitative polymerase chain reaction (qPCR), miRNA microarray, or bioinformatics-driven analysis where correlations are drawn between levels of a particular miRNA and either improved or worsened prognosis for GBM patients. An example is miR-517c, a member of the C19MC RNA cluster that has been shown to be positively correlated with improved prognosis in GBM patients [122]. The proposed mechanism of action is the miR-517c/karyopherin alpha 2 (KPNA2, RAG cohort 1, or importin alpha 1)/cytoplasmic p53 axis, where miR-517c degrades KPNA2, negatively impacting the nuclear translocation of p53, leading to the inhibition of autophagy in U87MG cells harboring wild-type p53. In the context of combination treatment with TMZ, inhibition of autophagy by miR-517c was correlated with reduced migration and infiltration, as well as an increased expression of epithelial markers and inhibition of endothelial-to-mesenchymal transition (EMT) [122].

Other groups found that overexpression of miRNA-30a and miRNA-17 led to the inhibition of autophagy and increased TMZ sensitivity in GBM [123,124]. The observation that TMZ treatment leads to a reduction of miRNA-30a levels in a dose-dependent manner has led investigators to explore the effects of overexpression of miRNA-30a, finding that miRNA-30a directly acts upon Beclin 1 and thus is an inhibitor of cytoprotective autophagy [124]. Moreover, evidence that miRNA-17 inhibits ATG7, an integral protein in autophagosome formation, motivated the investigators to transfect the miRNA into human GBM T98G cells and yield increased TMZ sensitivity [123]. In addition, overexpression of yet another miRNA, miR-7-1-3p, in concert with flavonoid combination therapy employing luteolin (LUT) and silibinin (SIL), led to autophagy inhibition in rapamycin (an mTOR inhibitor and promoter of autophagy) pre-treated U87MG and T98G tumors in vivo [125]. Although this study did not evaluate the effect of TMZ and miR-7-1-3p combination therapy, they found a synergistic combination of concentrations of LUT and SIL that yielded higher efficacy than TMZ monotherapy [125]. Conversely, overexpression of miR-138 led to upregulation of autophagy and TMZ resistance, with the proposed mechanism of action being the miR-138/Bim axis, where Bim is an upstream inhibitor of Beclin 1 [126,127].

Hypoxia is intricately linked to the promotion of cytoprotective autophagy in GBM, and specific miRNAs have been identified that participate in the pathways that link hypoxia and initiation of autophagy. A relationship between an increase in levels of HIF-1α and a decrease in levels of miR-224-3p led to the elucidation of the negative regulation of ATG5 due to the action of miR-224-3p [128]. As such, overexpression of miR-224-3p led to inhibition of autophagy, decreased cell mobility, and increased chemosensitivity of GBM cells to TMZ in hypoxic conditions [128]. Hypoxia has also been shown to induce the expression of interleukin-6 (IL-6), which has been correlated with a poor prognosis in GBM. Utilizing a pathway that involves miR-155-3p, IL-6 promotes autophagy, fueling the progression of GBM [129]. Specifically, the mechanism of action involves a hypoxia-induced IL-6/pSTAT3/miR-155-3p/cAMP responsive element binding protein 3 (CREB3)/ATG5 axis. Reduction in miR-155-3p led to inhibition of IL-6-induced cytoprotective autophagy and blockage of the IL-6 receptor; using tocilizumab in combination with TMZ showed drug synergism and elevated induction of apoptosis in human GBM U251 and T98G cell lines [129].

Identifying miRNA targets in GSCs is especially important as GSCs are notorious for promoting tumor recurrence and evading many therapeutics. One miRNA of note is miR-93, which inhibits multiple targets integral to autophagy promotion, including Beclin 1, ATG5, and ATG4B. Decreased miR-93 expression following radiotherapy, chemotherapy and rapamycin treatment in GSCs motivated investigation on the effects of ectopic expression of miR-93 [130]. Overexpression of miR-93 resulted in autophagy inhibition and GSC sensitization to radiotherapy and TMZ therapy [130]. Another ncRNA that engages in the regulation of both stemness and autophagy is the lncRNA metastasis-associated lung adenocarcinoma transcript 1 (MALAT1). MALAT1 is a tumor promoter involved in the maintenance of stemness, acting upon the Wnt pathway, SRY (sex-determining region Y)-related HMG (high mobility group)-box transcription factor 2 (SOX2), and Nestin [131]. Moreover, MALAT1 acts as a miRNA sponge, inhibiting miR-101 and inducing cytoprotective autophagy while suppressing apoptosis [132]. Additional suppression of miR-203 has connected MALAT1 to the decreased TMZ sensitivity via the promotion of EMT [133].

Identification of miRNA targets that modify autophagy pathways and that may be correlated with either improved or worsened prognosis in GBM has led to their exploitation for potentiation of TMZ action (Table 4). These miRNAs show potential for use in combination therapy to address current limitations such as hypoxia-induced cytoprotective autophagy; however, complex mechanisms of their action warrant additional investigation to understand potential targets in these miRNAs and reduce off-target effects.

## 7. Conclusions and Future Directions

GBM continues to be a highly deadly malignancy with a dismal prognosis even today. The discovery that concomitant and adjuvant administration of TMZ with radiotherapy could lead to significant regression of the disease and improvements in overall survival (OS) and progression-free survival (PFS) was a major advancement in GBM therapy [5]. However, several limitations still exist, as lack of MGMT promoter methylation renders TMZ largely ineffective, and the activation of cytoprotective autophagy in response to TMZ administration can power GBM chemotherapy resistance and tumor recurrence over time [8,12].

The homeostatic process that autophagy plays has a significant role in GBM progression. Cytoprotective autophagy activation can confer radiotherapy and chemotherapy resistance and enable cells to persist even in the hypoxic, nutrient-deprived TME. Through the degradation of cellular damaged molecules and organelles to generate basic building blocks that are the precursors for the TCA or the Krebs cycle and that sustain mitochondrial metabolism, autophagy promotes GBM growth potential and maintains GSC stemness [18,24]. Induction of autophagy following TMZ administration is often to counteract a state of stress and can lead to avoidance of apoptosis, induction of senescence, and an increase in levels of ATP for maintenance of cellular bioenergetics [52,53]. In addition, TMZ-induced autophagy plays a role in the maintenance of stemness in GSCs, with the exact targets of the autophagy pathway involved in either the upregulation or downregulation of stem cell markers and EMT still requiring additional investigation to elucidate their off-target effects [58,59].

To identify novel targets for the regulation of autophagy and mitigation of GBM progression, various advanced bioinformatics-driven approaches have been employed in recent years. Utilizing public genomic databases such as the TCGA, REMBRANDT, Gravendeel, KEGG, and CGGA, univariate and multivariate Cox regression and LASSO regression have been applied to both identify therapeutic targets and create predictive nomograms [61,62]. The accuracy of these nomograms seems to hover around an AUC value of 0.6 or 0.7 upon verification of the models, calling for additional datasets with expanded sample size and standardization of data analysis for greater reproducibility and opportunity for comparison among multiple studies. Finding correlations between expression levels of certain genes and prognosis in GBM could lead to the discovery of exciting new targets that could be exploited to inhibit autophagy and potentiate the action of TMZ for increasing the induction of apoptosis.

A breakthrough method to identify the action of and manipulate individual genes to counteract cancer progression and better model disease is CRISPR-Cas9 gene editing technology. Used in conjunction with bioinformatics-driven approaches, CRISPR-Cas9 gene editing technology can knockout or knock-in genes of interest to inhibit autophagy, GSC proliferation and mitigate immunosuppression. Various targets have been exploited in both the contexts of autophagy and GBM; however, an investigation into the modification of ARGs for GBM therapy, specifically in conjunction with TMZ combination therapy, is still limited [13]. Moreover, certain limitations with the delivery of CRISPR-Cas9 protein continue to persist, and as such, shorter variants such as *Cambpylobacter jejuni* Cas9 (CjCas9) are being explored; however, smaller proteins tend to require more complex protospacer adjacent motif (PAM) sequences, making them less practical when it comes to flexibility of genome targeting [83]. Another active area of investigation is addressing immunogenicity and the pre-existing humoral and cell-mediated adaptive immune responses to Cas9 proteins that some people may exhibit [83].

Targeting the genome with CRISPR-Cas9 technology is a highly effective method for inducing precise changes in gene expression; however, exploiting miRNAs can also lead to broad changes in protein expression and inhibition of cytoprotective autophagy in the context of combination therapy with TMZ in GBM. Identification of miRNA targets utilizing qPCR, miRNA microarrays, and bioinformatics-driven analysis has led to either their overexpression or knockdown to inhibit autophagy, synergize with TMZ, and induce apoptosis in GBM cell lines [123,124,128]. In addition, miRNAs that manipulate GSC proliferation have been identified with respect to their role in the inhibition of autophagy and have been investigated in the context of combination therapy with TMZ, showing promising therapeutic outcomes [130]. Ultimately, manipulation of miRNAs could prove effective in combination therapy, potentially also playing a role in the diagnosis of GBM. Understanding their mechanisms of action and targets is necessary for further investigation and for miRNAs to be leveraged to play a role in these synergistic therapeutic combinations in the future.

Other modern biology technologies continue to be developed for the inhibition of autophagy in GBM. Computational modeling has enabled the development and characterization of novel powerful small-molecular inhibitors. Examples include homology modeling to determine the structure of protein complexes and screening of libraries such as the Specs SC small molecule library for the identification of appropriate candidates for therapeutic purposes in GBM [134,135,136]. The creation of such libraries, the development of computational modeling, and the combination of these novel techniques with those described in this review article reveal a hopeful future for the targeted therapeutics in GBM.

GBM is a complex disease characterized by inter-tumoral and intra-tumoral heterogeneity, recurrence, and poor prognosis; however, many new advancements in both the identification of targets and development of novel genetic therapeutic strategies may lead to another breakthrough therapy that can supplement the current Stupp protocol to give GBM patients a new fighting chance.

## Figures and Tables

**Table 1:** Combination therapy with TMZ for targeting autophagy in clinical trials.

Drug Combined with TMZ	Role in Autophagy	Phase	Dosage	OS and PFS Clinical Outcomes	Adverse Effects	References
HCQ	Late-stage inhibitor	I	200–1200 mg/day	Partial response in melanoma	Fatigue, anorexia, nausea, constipation, and diarrhea	[46]
		I/II	600 mg/day (MTD)	MS of 15.6 months in GBM	Grade 3 and 4 neutropenia and thrombocytopenia at 800 mg/day HCQ	[47]
CQ	Late-stage inhibitor	I	150 mg/day	MS of 24 months in GBM	Seizures due to neoplasm; no other adverse effects	[48]
		II	400 mg/day	Not yet recruiting	NA	[49]
TN-TC11G (THC-CBD)	Promoter via TRB3 pathway	IB	5–40 mg/3 times/day	Not yet recruiting	NA	[50]
Bortezomib	Inhibitor	IB/II	1.3 mg/m^2^/3 times/week	Currently recruiting	NA	[51]

HCQ, hydroxychloroquine; CQ, chloroquine; MTD, maximum tolerated dose; RT, radiotherapy; OS, overall survival; PFS, progression-free survival; MS, median survival; NA, not available; NLM, National Library of Medicine; and TRB3, tribbles-related protein 3.

**Table 4:** miRNAs that modulate autophagy to potentiate TMZ treatment in GBM.

miRNA	Mechanism of Action	Results	References
miR-517c	miR-517c/KPNA2/cytoplasmic p53 axis; inhibition of autophagy in wild type-p53 U87MG cell line	Combination with TMZ reduced migration and infiltration, increased expression of epithelial markers and inhibition of EMT	[122]
miR-17	ATG7 inhibitor	Increased TMZ sensitivity and cell death	[123]
miR-30a	Beclin 1 inhibitor	TMZ treatment downregulated miR-30a in a dose-dependent manner	[124]
miR-7-1-3p	Targets BIRC4 to promote apoptosis	Combination with SIL and LUT led to autophagy inhibition in rapamycin-treated GBM cell lines	[125]
miR-138	miR-138/Bim axis; upstream promotion of Beclin 1	Upregulation led to promotion of autophagy and TMZ resistance	[126,127]
miR-224-3p	Downregulation of ATG5	Inhibition of autophagy, decreased cell mobility, increased sensitivity to TMZ	[128]
miR-155-3p	Hypoxia-induced IL-6/pSTAT3/miR-155-3p/CREB3/ATG5 axis	KD of miRNA led to inhibition of IL-6-induced cytoprotective autophagy; synergism with TMZ therapy	[129]
miR-93	Beclin 1, ATG5, and ATG4B inhibitor	Overexpression led to autophagy inhibition and GSC sensitization to TMZ therapy	[130]
MALAT1 (lncRNA)	miR-101 and miR-203 inhibitor	Maintained stemness and induced protective autophagy; decreased TMZ sensitivity due to an increase in EMT	[131,132,133]
